# Housing Status and Acute Care Use After Cancer Diagnosis

**DOI:** 10.1001/jamanetworkopen.2024.19657

**Published:** 2024-07-02

**Authors:** Hannah Decker, Sara Colom, Dave Graham-Squire, Elizabeth Wick, Margot B. Kushel, Maria Raven, Hemal K. Kanzaria

**Affiliations:** 1 Department of Surgery, University of California, San Francisco; 2 Benioff Homelessness and Housing Initiative, Zuckerberg San Francisco General Hospital, California; 3 Department of Internal Medicine, University of California, San Francisco; 4 Department of Emergency Medicine, University of California, San Francisco

## Abstract

This cohort study examines housing status and acute care use after a cancer diagnosis among individuals treated at a public hospital in San Francisco, California.

## Introduction

Homelessness is associated with poor outcomes following cancer diagnosis due, in part, to later-stage diagnoses and inadequate access to guideline-concordant therapy, which requires coordinated, multidisciplinary communication among clinicians and patients.^1,2^ Acute care use following cancer diagnosis may signal challenges associated with accessing scheduled oncologic treatment, symptom management, or care for comorbidities.^3,4^ We hypothesized that individuals who were unhoused would have greater increases in acute care use after cancer diagnosis compared with individuals who were housed.

## Methods

This cohort study followed the Strengthening the Reporting of Observational Studies in Epidemiology (STROBE) reporting guideline and was approved by the University of California San Francisco institutional review board. Informed consent was waived because of the retrospective nature of the study.

We conducted a retrospective cohort study examining the association between housing status and acute care use in adult patients treated for cancer at Zuckerberg San Francisco General Hospital (ZSFG), a public hospital for San Francisco City and County, from July 1, 2011, to June 30, 2021. We merged the ZSFG Cancer Registry with the Coordinated Care Management System (CCMS), an integrated San Francisco Department of Public Health data system that links information about use of county social services and physical and behavioral health care (eMethods in Supplement 1).^5^

Our exposure was housing status, stratified into unhoused, formerly unhoused (but not in the year of cancer diagnosis), and housed. CCMS tracks both observed (eg, housing navigation services, shelter use) and reported (eg, during a clinical encounter) homelessness. Our primary outcome was the absolute change in counts of acute care use (emergency department [ED] visits, hospital stays and psychiatric emergency services [PES] visits) from the year before to the year of cancer diagnosis.

We performed bivariate comparisons between housing status groups and multivariable linear regression with robust standard errors to examine if housing status was associated with a change in acute care use. We adjusted for sex, age, cancer stage, cancer site, year of diagnosis, race, ethnicity, marital status, smoking, alcohol use, and Elixhauser Comorbidity score (eMethods in Supplement 1). Data were analyzed from July 2023 to April 2024. Statistical tests were 2-sided, and significance was set as *P* < .05. Analyses were performed in Stata version 18 (StataCorp).

## Result

Our cohort included 3827 people, of which 2139 (56%) were males, 838 (22%) were Black individuals, and 675 (18%) were Latinx individuals. The median (IQR) age at cancer diagnosis was 61 (54-68) years. The most common diagnoses were lung, breast, and colorectal cancers; 438 (11%) of the cohort were unhoused and 623 (16%) were formerly homeless (Table 1).

**Table 1.** Clinical and Demographic Characteristics of Cancer Diagnoses, by Housing Status

Characteristics	Participants, No. (%)			*P* value^a^
	Housed (n = 2766)	Formerly unhoused (n = 623)	Unhoused (n = 438)	
Age at diagnosis, median (IQR)	62 (54-69)	60 (55-66)	58 (51-64)	<.001
Sex				
Male	1357 (49.1)	446 (71.6)	336 (76.7)	<.001
Female	1405 (50.8)	169 (27.1)	(98) (22.4)	
Race				
Asian or Pacific Islander^b^	1182 (42.7)	34 (5.5)	26 (5.9)	<.001
Black	370 (13.4)	284 (45.6)	42.0 (184)	
White	1173 (42.4)	296 (47.5)	223 (50.9)	
Other^c^	41 (1.5)	9 (1.4)	1.1 (5) (1.1)	
Marital status				
Married or domestic partner	1031 (37.3)	47 (7.5)	24 (5.5)	<.001
Single, separated, divorced, widowed	1523 (55.1)	514 (82.5)	357 (81.5)	
Elixhauser score, median (IQR)	9 (0-19)	15 (4-24)	9 (0-21)	<.001
Site^d^				
Lung	385 (13.9)	107 (17.2)	75 (17.1)	<.001
Breast	305 (11.0)	30 (4.8)	26 (5.9)	
Colorectal	284 (10.3)	46 (7.4)	28 (6.4)	
Liver and biliary tract	214 (7.7)	90 (14.4)	57 (13.0)	
Kidneys, ureter, bladder, and other urinary tract	205 (7.4)	53 (8.5)	42 (9.6)	
Female reproductive tract	205 (7.4)	23 (3.7)	13 (3.0)	
Prostate	122 (4.4)	44 (7.1)	36 (8.2)	
Head and neck	129 (4.7)	64 (10.3)	6.4 (28)	
Nervous system, including brain	148 (5.4)	21 (3.4)	14 (3.2)	
Blood or bone marrow	111 (4.0)	22 (3.5)	20 (4.6)	
Lymph nodes	91 (3.3)	19 (3.0)	16 (3.7)	
Skin	56 (2.0)	12 (1.9)	19 (4.3)	
Thyroid	95 (3.4)	2 (0.3)	11 (2.5)	
Stomach	88 (3.2)	12 (1.9)	9 (2.1)	
Pancreas	77 (2.8)	19 (3.0)	2 (0.5)	
Other	251 (9.1)	59 (9.5)	42 (9.6)	
Stage at diagnosis				
0	126 (4.6)	25 (4.0)	13 (3.0)	.41
I	727 (26.3)	165 (26.5)	119 (27.2)	
II	340 (12.3)	85 (13.6)	58 (13.2)	
III	390 (14.1)	81 (13.0)	48 (11.0)	
IV	677 (24.5)	166 (26.6)	124 (28.3)	
Missing	506 (18.3)	101 (16.2)	76 (17.4)	

^a^ χ^2^ tests were used for categorical variables and Kruskal-Wallis tests were used for continuous variables.

^b^ Asian or Pacific Islander includes: Chinese, Japanese, Filipino, Korean, Vietnamese, Laotian, Kampuchean, Thai, Asian Indian, Samoan, Tongan, Fiji Islander, Other South Asian, Asian not otherwise specified, Pacific Islander not otherwise specified.

^c^ Other includes: Other, Unknown, American Indian, Hawaiian, Pakistani.

^d^ Male predominance of the unhoused population is likely driving the difference in sex-related cancer sites (breast, female reproductive tract).

Baseline acute care use was high among patients who were unhoused the year before cancer diagnosis, with 71 of 438 (16%) having 4 or more ED visits (vs 70 of 2766 [3%] of individuals who were housed and 58 of 623 [9%] of individuals who were formerly unhoused). The number of ED visits and hospital stays increased in all housing status groups from the year before to the year of cancer diagnosis but increased disproportionately in unhoused patients compared with housed; this finding persisted even after adjusting for clinical and demographic covariates (adjusted coefficient for ED visits, 0.78; 95% CI, 0.16-1.40; *P* = .01; and hospitalizations, 0.33; 95% CI, 0.08-0.57; *P* = .009) (Table 2). PES visits did not change significantly among any housing status group.

**Table 2.** Association of Housing Status With Change in Acute Care Use From the Year Before to the Year of Cancer Diagnosis

Housing category	Unadjusted coefficient (95% CI)	*P* value	Adjusted coefficient (95% CI)^a^	*P* value
**ED visits**				
Housed	[Reference]	NA	[Reference]	NA
Formerly unhoused	0.42 (0.17 to 0.66)	.001	−0.34 (−0.70 to 0.01)	.06
Unhoused	1.59 (0.99 to −2.19)	<.001	0.78 (0.16 to 1.40)	.01
**Hospitalizations**				
Housed	[Reference]	NA	[Reference]	NA
Formerly unhoused	0.34 (0.21 to 0.47)	<.001	−0.14 (−0.32 to 0.03)	.10
Unhoused	0.77 (0.57 to 0.97)	<.001	0.33 (0.08 to 0.57)	.009
**PES visits**				
Housed	[Reference]	NA	[Reference]	NA
Formerly unhoused	0.004 (−0.02 to 0.02)	.73	−0.01 (−0.04 to 0.02)	.67
Unhoused	−0.13 (−0.14 to 0.11)	.84	0.04 (−0.04 to 0.12)	.32

Abbreviations: ED, emergency department; NA, not applicable; PES, psychiatric emergency services.

^a^ Adjusted for sex, age, cancer stage, cancer site, year of diagnosis, race, ethnicity, marital status, smoking, alcohol use, and Elixhauser Comorbidity score.

## Discussion

Among patients receiving treatment for cancer at an urban public hospital, acute care use increased disproportionately in unhoused patients in the year of diagnosis, while psychiatric acute care use did not change. This finding may reflect homelessness disrupting access to traditional outpatient pathways for managing cancer, adding complexity to already challenging care coordination needs in this population. This dynamic may contribute to delays in cancer-directed therapy and to the poor outcomes seen in unhoused patients compared with the general population with cancer. This study is limited because it only examines individuals with cancer who are linked to CCMS, representing a high-use, complex population which may limit external generalizability. Further research should investigate how best to meet the needs of patients who were unhoused with cancer and consider methods to engage acute care clinicians in contingency planning and coordination with oncologic clinicians.

## Notes

Supplement 1.
eMethods.

Supplement 2.
Data Sharing Statement
