# Supplementary material for: Housing Status and Acute Care Use After Cancer Diagnosis
Source: JAMA Netw Open. 2024 Jul 2;7(7):e2419657. doi: 10.1001/jamanetworkopen.2024.19657 (PMC11220561; doi:10.1001/jamanetworkopen.2024.19657)
Supplement: Supplement 1. — eMethods. [file jamanetwopen-e2419657-s001.pdf]

Supplemental Online Content

Decker HC, Colom Brana S, Graham-Squire D, et al. Housing status and acute care use after cancer diagnosis. *JAMA Netw Open*. 2024;7(7):e2419657. doi:10.1001/jamanetworkopen.2024.19657

eMethods.

This supplemental material has been provided by the authors to give readers additional information about their work.

© 2024 Decker HC et al. *JAMA Network Open.*

eMethods.

Description of the ZSFG Cancer Registry

The ZSFG Cancer Registry includes all patients who were diagnosed or received any treatment at ZSFG. Cancer Registry personnel report cases to the registry with chart abstraction of key clinical and oncologic details, regular external quality audits, and clinician oversight of key details.

Description of Coordinated Care Management System (CCMS)

CCMS is an integrated data system hosted by the San Francisco Department of Public Health (DPH) that links information about service use across mental health, physical health, substance use, and social health domains in a vulnerable, complex, and high-risk population.

CCMS includes information on both in-network and out-of-network emergency department visits and hospital stays for San Francisco Health Plan (SFHP) beneficiaries (which comprise 60% of individuals captured in CCMS). For non-SFHP beneficiaries, only in-network ED and hospital use is captured.

Description of CCMS Record Creation

A CCMS record is created for any patient observed or reported to be unhoused in a DPH or county housing system. Reports can come from clinical encounters (with behavioral, substance use, and physical health providers) or engagement with social services (including shelters, housing navigation, coordinated entry, among others). This reporting leads to a more thorough capture of unhoused patients than using the cancer registry alone (which identified only 30% of the total unhoused cohort as unhoused) or zip codes, as the 438 unhoused individuals reported 44 unique zip codes, (the most common of which was the zip code of the hospital).

CCMS records are also created for any person who has engaged with county behavioral health or jail services, or engaged with urgent/emergent services across physical health, mental health, substance use, or social health domains.

Covariates

We obtained information on participant race and ethnicity from the ZSFG Cancer Registry, which is primarily based on self-report. We evaluated this information because of data highlighting inequities in care utilization and cancer outcomes based on race and ethnicity.

Statistical Detail

For this analysis, we restricted our analytic sample to only individuals in the ZSFG Cancer Registry who were also identified in CCMS (75% of the total cancer diagnoses) because we only had information about acute care utilization for this subset.

Linear Regression

We used robust standard errors to account for minor deviations in normality in the residuals of the predicted values in the linear regression.

© 2024 Decker HC et al. *JAMA Network Open.*

Sensitivity Analysis
As a sensitivity analysis, we also constructed generalized estimating equation models, clustering on the patient, with a negative binomial distribution, a log link function, an exchangeable correlation structure and robust standard errors as a repeated measures analysis. We constructed three models, with the outcomes being: 1) Counts of ED visits, 2) Counts of Admissions and 3) Counts of PES visits. For each, our exposure was housing status. We adjusted for period (that is, fiscal year before or fiscal year of cancer diagnosis) as well as an interaction term between housing status and period. We also adjusted for the same covariates as in the original linear regression model (age at diagnosis, site, race, marital status, smoking, alcohol use, stage, ethnicity, Elixhauser score, sex, and year of diagnosis) in each model. As these models yielded similar results to the linear regression models, we present the former in the paper.

Additional References

- Kanzaria HK, Niedzwiecki M, Cawley CL, et al. Frequent Emergency Department Users: Focusing Solely On Medical Utilization Misses The Whole Person. Health Aff (Millwood). 2019;38(11):1866-1875. doi:10.1377/HLTHAFF.2019.00082

- Molina M, Evans J, Montoy JC, et al. Analysis of Emergency Department Encounters Among High Users of Health Care and Social Service Systems Before and During the COVID-19 Pandemic. *JAMA Netw Open.* 2022;5(10):e2239076. doi:10.1001/jamanetworkopen.2022.39076

- Cawley C, Henderson J, Kanzaria HK, Lacoe J, Paolillo S, Perez K, Raven M, Skog A. Signals of High Utilization of Criminal Legal and Urgent and Emergent Health Services in San Francisco. California Policy Lab (2022). https://www.capolicylab.org/signals-of-distress-high-utilization-of-criminal-legal-and-urgent-and-emergent-health-services-in-san-francisco/. Accessed December 27, 2023.

- Hewlett MM, Raven MC, Graham-Squire D, Evans JL, Cawley C, Kushel M, Kanzaria HK. Cluster Analysis of the Highest Users of Medical, Behavioral Health, and Social Services in San Francisco. J Gen Intern Med. 2023 Apr;38(5):1143-1151. doi: 10.1007/s11606-022-07873-y. Epub 2022 Nov 29. PMID: 36447066; PMCID: PMC9708142.

© 2024 Decker HC et al. *JAMA Network Open.*
