# Supplementary material for: Housing Status and Acute Care Use After Cancer Diagnosis
Source: JAMA Netw Open. 2024 Jul 2;7(7):e2419657. doi: 10.1001/jamanetworkopen.2024.19657 (PMC11220561; doi:10.1001/jamanetworkopen.2024.19657)
Supplement: Supplement 2. — Data Sharing Statement [file jamanetwopen-e2419657-s002.pdf]

Data Sharing Statement

Decker. Housing Status and Acute Care Use After Cancer Diagnosis. *JAMA Netw Open*.
Published July 02, 2024. doi:10.1001/jamanetworkopen.2024.19657

Data
Data available: Yes
Data types: Data dictionary
How to access data: Please email Hannah.decker@ucsf.edu.
When available: With publication

Supporting Documents
Document types: None

Additional Information
Who can access the data: Researchers for whom research proposal has been approved.
Types of analyses: For approved research.
Mechanisms of data availability: After approval of a proposal.
